# Spectrum of cardiac lesions associated with Isolated Cleft Mitral Valve and their impact on therapeutic choices

**DOI:** 10.5935/abc.20160053

**Published:** 2016-05

**Authors:** Ayoub El hammiri, Abdenasser Drighil, Sanaa Benhaourech

**Affiliations:** Cardiology Department, Ibn Rochd University Hospital, Casablanca, Morocco

**Keywords:** Heart Defects, Congenital / complications, Heart Valve Diseases / surgery, Mitral Valve Insufficiency / complications

## Abstract

**Background:**

Isolated cleft mitral valve (ICMV) may occur alone or in association with other congenital heart lesions. The aim of this study was to describe the profile of cardiac lesions associated with ICMV and their potential impact on therapeutic management.

**Methods:**

We conducted a descriptive study with data retrieved from the Congenital Heart Disease (CHD) single-center registry of our institution, including patients with ICMV registered between December 2008 and November 2014.

**Results:**

Among 2177 patients retrieved from the CHD registry, 22 (1%) had ICMV. Median age at diagnosis was 5 years (6 days to 36 years). Nine patients (40.9%) had Down syndrome. Seventeen patients (77.3%) had associated lesions, including 11 (64.7%) with accessory chordae in the left ventricular outflow tract (LVOT) with no obstruction, 15 (88.2%) had ventricular septal defect (VSD), three had secundum atrial septal defect, and four had patent ductus arteriosus. Thirteen patients (59.1%) required surgical repair. The decision to proceed with surgery was mainly based on the severity of the associated lesion in eight patients (61.5%) and on the severity of the mitral regurgitation in four patients (30.8%). In one patient, surgery was decided based on the severity of both the associated lesion and mitral regurgitation.

**Conclusion:**

Our study shows that ICMV is rare and strongly associated with Down syndrome. The most common associated cardiac abnormalities were VSD and accessory chordae in the LVOT. We conclude that cardiac lesions associated with ICMV are of major interest, since in this study patients with cardiac lesions were diagnosed earlier. The decision to operate on these patients must take into account the severity of both mitral regurgitation and associated cardiac lesions.

## Introduction

A mitral valve cleft not associated with an atrioventricular septal defect (AVSD) or a common atrioventricular junction is often referred as "isolated cleft mitral valve" (ICMV) or as "true mitral valve cleft". This uncommon congenital cardiac disease is associated with a variable degree of mitral regurgitation (MR) and may occur alone or in association with other congenital heart lesions.

Some earlier reports have highlighted the anatomical and echocardiographic features of ICMV.^1-6^ With the advent of high-resolution echocardiography, ICMV and associated cardiac lesions may be readily diagnosed.^2,3^ Recognition of the spectrum of cardiac abnormalities associated with ICMV is paramount, as the severity of the associated cardiac lesions may determine the timing of the intervention. Patients with associated lesions have been reported to be more symptomatic and, therefore, tend to be diagnosed earlier than patients with ICMV alone. Also, the type and severity of the associated cardiac lesions may influence the type of intervention in the setting of substantial MR.

The aim of our study was to identify the spectrum of the cardiac lesions associated with ICMV and define their impact on the diagnosis delay and the therapeutic management.

## Methods

Our study used data from patients registered from December 2008 to November 2014 obtained from the Congenital Heart Disease (CHD) single-center registry of our university hospital. The diagnosis of ICMV was based on echocardiographic findings in all patients and was established by the same operator (A.D.). ICMV was defined as the presence of a cleft in the anterior leaflet of the mitral valve, visualized in the parasternal short axis or subcostal view of the mitral valve. Consistent with Anderson's description,^7^ the cleft should appear very much like an "artificial cleft produced in a normal valve with a scalpel".

Definitions of the positions of the cleft as "anterior lateral" or "anterior median" was based on the description by Di Segni et al.^3^ Anterior median refers to the classic position of the cleft, as described by the authors, in the middle of the anterior leaflet, pointing toward the left ventricular outflow tract (LVOT). Anterior lateral, in turn, refers to decentralized clefts, in which the lateral portion of the anterior leaflet is wider than the medial portion or the medial portion is wider.

Demographic and clinical characteristics, as well as therapeutic management of all patient with ICMV were recorded. Patients with AVSD were excluded from the analysis. The severity of the MR was estimated qualitatively by color Doppler with the evaluation of the degree of regurgitant mitral flow in the left atrium (LA), and/or quantitatively using the proximal isovelocity surface area to calculate the effective regurgitant orifice area (EROA) with the formula $\mathrm{EROA}=\frac{2\pi r^{2}\mathrm{Nyquist}\;\mathrm{Limit}}{V\max}\times \frac{a}{180}$ or with a simplified formula $(\mathrm{EROA}=\frac{r^{2}}{2})$.^8^ The degree of MR was classified as follows: (1) mild, when the color flow of the MR jet was small and central (usually 4 cm^2^ or < 20% of the LA area), and/or EROA < 0.20 cm^2^, regurgitant volume (RV) < 30 mL, regurgitant fraction (RF) < 30%; (2) moderate, when the color flow of the MR jet was intermediate and/or 0.20 ≤ EROA < 0.40 cm^2^, 30 ≤ RV < 60 mL, 30% ≤ RF < 50%; and (3) severe, when the color flow of the MR jet was large and central (usually >10 cm^2^ or > 40% of the LA area) or in the presence of a wall-impinging jet swirling of variable size in the LA and/or EROA ≥ 0.40 cm^2^, RV ≥ 60 mL, RF ≥ 50%.^9,10^

The systolic pulmonary artery pressure (SPAP) was estimated from the tricuspid regurgitant jet peak velocity using the modified Bernoulli equation (peak gradient = 4V^2^, where V is the maximal velocity of the tricuspid regurgitant jet measured by continuous-wave Doppler).

We reported all associated cardiac lesions and described them according to the international nomenclature based on the International Pediatric and Congenital Cardiac Code.^11^ SA peak Doppler gradient greater than 20 mmHg characterized the occurrence of a subaortic obstruction.

### Statistical analysis

Data are expressed as mean ± standard deviation or as median (range), and categorical variables are expressed as percentage. All analyses were conducted using SPSS (version 20.0, SPSS Inc., Chicago, IL, USA).

## Results

Among 2177 patients with a CHD, 22 (1%) had an ICMV. The median age at diagnosis was 5 years (6 days to 36 years), and 50% of the patients were diagnosed before the age of 4 years. Patients with associated lesions were diagnosed at a median age of 20 months (range 6 days to 33 years), and patients without associated lesions were diagnosed at a median age of 9 years (range 6 years to 36 years). The rate of consanguinity was 9.1%, and the male-to-female ratio was 0.83. Nine patients (40.9%) had Down syndrome. In one patient the cleft was in an anterior lateral position, and in 21 (95.5%) patients the cleft was in the an anterior median position. MR was severe in 5 patients (22.7%), moderate in two patients (9.1%), mild in six patients (27.3%), and absent in nine patients (40.9%) (Table 1).

**Table 1:** General characteristics of the ICMV population

**General characteristics**	**ICMV associated with other cardiac lesions**	**ICMV alone**	**All patients**
Number of patients	17 (77.3%)	5 (22.7%)	22 (100%)
Median age at diagnosis	2 years and 9 months (6 days to 33 years)	12 years (9 years to 36 years)	5 years (6 days to 36 years)
Consanguinity rate	2 (11.8%)	0	2 (9.1%)
**Gender**			
Male	7 (41.2%)	3 (60%)	10 (45.5%)
Female	10 (58.8%)	2 (40%)	12 (54.5%)
Ratio	0.7	1.5	0.8
Down syndrome	9 (52.9%)	0	9 (40.9%)
**Location of the cleft**			
Anterior median	17 (100%)	4 (80%)	21 (95.5%)
Anterior lateral	0	1 (20%)	1 (5.5%)
**Level of regurgitation**			
Severe	2 (11.8%)	3 (60%)	5 (22.7%)
Moderate	2 (11.8%)	0	2 (9.1%)
Mild	4 (23.5%)	2 (40%)	6 (27.3%)
Absentusente	9 (52.9%)	0	9 (40.9%)
**Therapeutic management**			
Surgical	11 (64.7%)	2 (40%)	13 (59.1%)
Medical	4 (23.5%)	2 (40%)	6 (27.3%)
None	2 (11.8%)	1 (20%)	3 (13.6%)

ICMV: isolated cleft mitral valve.

**Figure 1:**
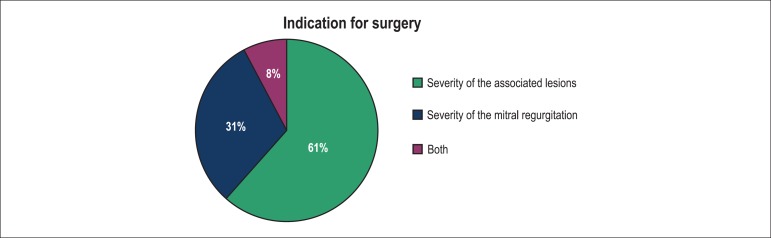
Reasons for choosing surgery as a therapeutic option in a population with isolated cleft mitral valve.

**Figure 2:**
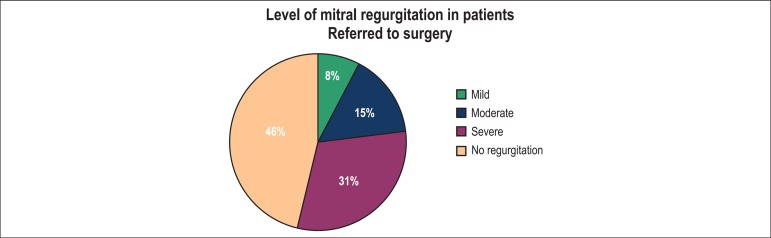
Level of mitral regurgitation in patients with isolated cleft mitral valve referred to surgery.

A total of 17 patients (77.3%) had associated lesions (Table 2). Among them, 11 (64.7%) had accessory chordae in the LVOT with no obstruction, 15 (88.2%) had ventricular septal defects (VSD; perimembranous = 8, inlet = 7) with a median diameter of 14 mm (2 mm to 41 mm), three patients had *secundum* atrial septal defects (ASD), and four patients had patent ductus arteriosus (PDA). Eight patients (36.4%) had pulmonary hypertension. Surgery was proposed to 13 patients, the recommendations was based on the severity of the associated lesions in eight patients (61.5%) and the severity of the MR in four patients (30.8%) (Figures 1 and 2). In one patient, the decision of surgery was based on the severity of both the associated lesion and mitral regurgitation.

**Table 2:** Distribution of associated cardiac lesions in ICMV

**Type of associated lesion**	**Number and percentage (n = 17)**
VSD	15 (88.2%)
Perimembranous	8 (47.1%)
Inlet	7 (41.2%)
Accessory chordae in LVOT	11 (64.7%)
PDA	4 (23.5%)
ASD *(ostium secundum)*	3 (17.7%)

ICMV: isolated cleft mitral valve; VSD: ventricular septal defect; LVOT: left ventricular outflow tract; PDA: patent ductus arteriosus; ASD: atrial septal defect.

## Discussion

The first description of ICMV was in 1954 by Edwards et al.^12^ The definition of ICMV by the Congenital Heart Surgery Nomenclature and Database Project is a "cleft in the anterior mitral valve leaflet not associated with *primum* ASD or other features of AVSD (with or without other associated defects)".^13^ It is a rare cause of congenital MR with an incidence in the pediatric population of 1:1340.^14^ In our study, ICMV represented 1% of all CHD.

As described by Perier and Clausnizer et al.,^15^ some anatomic features are specific to ICMV. Unlike clefts associated with AVSD, the mitral annulus in ICMV is located in its normal anatomic position and the cleft points towards the LVOT, whereas the mitral and tricuspid valves are attached to the interventricular septum at different levels (the tricuspid valve junction is lower than that of the mitral valve junction).

In our study, the age at diagnosis ranged from 6 days to 36 years, which is consistent with previous data reporting an age ranges from 1 day to 52 years.^1,3,15,16^ Patients with associated lesions were diagnosed at a median age of 20 months (range 6 days to 33 years), whereas those without associated lesions were diagnosed at a median age of 9 years (range 6 years to 36 years). These findings are consistent with a previous study^17^ showing that patients with ICMV and associated lesions had an earlier presentation and substantial cardiac symptoms, which related more to the associated cardiac lesions than to the ICMV itself.

In our study, 40.9% of the patients had Down syndrome. This frequency was higher in comparison with other published series that have reported incidences from 10% to 25%.^5,6,17^ In a recent study, Thankavel et al. reported an incidence of ICMV of 6.5% in individuals with the syndrome.^18^ This high incidence of Down syndrome in patients with ICMV suggests a possible connection between this genetic abnormality and ICMV.

Isolated clefts can be diagnosed adequately by two dimensional Doppler echocardiography.^19^ Following the advent of high-resolution cross-sectional echocardiography and three-dimensional echocardiography, ICMV has been more readily diagnosed and appears to be more common than previously thought.^4,20^ Echocardiography can demonstrate the cleft and evaluate its severity. In our series, MR was graded as moderate or severe in 31.8% of the cases, whereas ICMV without regurgitation was recorded in nine patients (40.9%). These findings contrast with those by Fraisse et al.,^21^ who reported a higher incidence of moderate to severe MR (45.5%). Furthermore, echocardiography may identify anatomical malformations associated with ICVM, such as *secundum* ASD, transposition of the great arteries, VSD, tricuspid atresia, PDA, coarctation of the aorta, double outlet right ventricle, and anomalous pulmonary venous connection.^22,23^ In our study, ICMV was associated with other congenital cardiac lesions in 17 patients (77.3%). The most frequent associated lesion was VSD in 15 patients (88.23%), which is in agreement with the findings by Zhu and al.^24^ who have reported an associated VSD in 50% of the patients with ICVM. Accessory chordae was found in 11 patients (64.7%), none of whom had LVOT obstruction. While an incomplete coaptation of the two halves of the cleft anterior leaflet is typically the cause of associated MR, particularly when a wide cleft is present,^6^ accessory chordae may cause LVOT stenosis and thereby contribute to the mechanism of MR. Previous pathological studies have also suggested that restriction of the motion of the anterior mitral leaflet caused by accessory chordae may be an additional mechanism.^6^ The importance of this latter mechanism has long been recognized as a limitation to the success of mitral valve repairs in patients with AVSD once the cleft has been sutured,^25^ but no data have been described addressing the impact of the existence of accessory chordae on the success of surgical repair in an ICMV population.

Of all patients referred to surgery in our study, 61% were referred because of the severity of the associated lesions. Only 31% of the patients were operated on exclusively due to the severity of the MR. A total of 54% of the patients operated on in our study had mild to moderate MR, suggesting that the indication for surgery was based on the severity and complexity of the associated lesions in most patients.

The high incidence of associated congenital cardiac lesions in the ICMV population is of major interest. Often, a substantial cleft can be repaired at the same time that the other cardiac lesions are repaired. In the absence of other congenital cardiac lesions, the decision about surgical intervention should be taken based on the MR severity and its clinical impact. If the degree of regurgitation is only mild to moderate, the surgical repair is not urgent, since the regurgitation does not progress over the intermediate term.^24^

## Conclusion

Our study shows that ICMV is rare and frequently associated with Down syndrome. Perimembranous VSD and accessory chordae in the LVOT were the most common associated cardiac abnormalities. ICMV may be of major interest in these cases because patients with associated lesions seem to be diagnosed earlier. The decision to operate on the ICMV should take into account the severity of both the MR and associated cardiac lesion.
